# The Study of Object-Oriented Motor Imagery Based on EEG Suppression

**DOI:** 10.1371/journal.pone.0144256

**Published:** 2015-12-07

**Authors:** Lili Li, Jing Wang, Guanghua Xu, Min Li, Jun Xie

**Affiliations:** 1 School of Mechanical Engineering, Xi’an Jiaotong University, Xi’an, Shaanxi, People’s Republic of China; 2 State Key Laboratory for Manufacturing Systems Engineering, Xi’an Jiaotong University, Xi’an, Shaanxi, People’s Republic of China; University of Electronic Science and Technology of China, CHINA

## Abstract

Motor imagery is a conventional method for brain computer interface and motor learning. To avoid the great individual difference of the motor imagery ability, object-oriented motor imagery was applied, and the effects were studied. Kinesthetic motor imagery and visual observation were administered to 15 healthy volunteers. The EEG during cue-based simple imagery (SI), object-oriented motor imagery (OI), non-object-oriented motor imagery (NI) and visual observation (VO) was recorded. Study results showed that OI and NI presented significant contralateral suppression in mu rhythm (*p* < 0.05). Besides, OI exhibited significant contralateral suppression in beta rhythm (*p* < 0.05). While no significant mu or beta contralateral suppression could be found during VO or SI (*p* > 0.05). Compared with NI, OI showed significant difference (*p* < 0.05) in mu rhythm and weak significant difference (*p* = 0.0612) in beta rhythm over the contralateral hemisphere. The ability of motor imagery can be reflected by the suppression degree of mu and beta frequencies which are the motor related rhythms. Thus, greater enhancement of activation in mirror neuron system is involved in response to object-oriented motor imagery. The object-oriented motor imagery is favorable for improvement of motor imagery ability.

## Introduction

Motor imagery (MI) is defined as a dynamic state during which the subject feels himself/herself performing an action. It is found that MI shares the similar cognitive processes [1] and sensory motor cortical [2] with movement execution. Therefore, MI is widely used for motor learning, such as in a variety of sports, e.g., rhythmic gymnastics [3], badminton [4] and slalom [5], and in rehabilitation of patients who cannot do active movements after a stroke [6], spinal cord injury [7], or Parkinson’s disease [8]. MI has also been a widely used strategy of brain computer interface (BCI) for controlling an unmanned aerial vehicle [9] or robot arm [10].

According to the simulation theory, MI involves a (partial) internal simulation of the seen or imagined action from the first person perspective [11]. MI is thought to be mediated by the mirror neuron system (MNS) [12]. The mirror neuron system is originally discovered in the premotor cortex of macaque (area F5) [13]. The research of the mirror neuron system elicited by electroencephalography (EEG) focuses on the modulation of rhythm, especially mu rhythms [14]. So far, there is agreement that the mu frequency oscillation in particular, which is a kind of motor related rhythm, is linked to the mirror neuron activity. Imagining motor actions can modulate the motor related rhythm and result in power changes. The power changes are known as event related desynchronization/synchronization (ERD/ERS), which has been suggested to be indicator of the ability of MI [2].

Although motor imagery is widely applied in motor learning, the performance of MI greatly depends on the mental imagery ability of subjects. This varies with their abilities to form a mental representation of an action. Moreover, the performance of MI also depends on the person’s attention to the mental imagery. Perceptual studies involving biological motion of a target may capture the attention of the subject more than non-target stimuli [14]. Chiviacowsky et al. [15] also state that performing a task could improve motor imagery ability compared to only directing attention to the body movements. In many rehabilitation studies, patients are guided to imagine object-oriented tasks, such as grasping an object [16,17]. It seems that imagining an object-oriented task could achieve better performance in rehabilitation.

This research aims to investigate the effects of biological object-oriented motor task on the motor imagery. That is, the research attends to study whether the presence of a biological object in the motor imagery can improve the vividness of kinesthetic imagery and generate greater changes on ERD/ERS. Results of this study might be beneficial to motor rehabilitation of lower extremities and motor skills training based on motor imagery.

## Materials and Methods

### Subjects and Recordings

The study included 15 right-handed participants consisting of 13 males and 2 females with a mean age of 23.5 ± 1.37 years. They were volunteers at Xi’an Jiaotong University. All participants had normal vision and motion abilities as well as an intact central nervous system. This study was approved by the institutional review board of Xi’an Jiaotong University. Subjects participated in the study after signing a written consent form. EEG data was recorded from 128 Ag-AgCl active electrodes with a g.HIamp (g.tec Inc., Austria) system according to the 10–5 international system [18]. EEG signals were referenced to a unilateral earlobe, grounded at a frontal position (Fpz). They were also online band-pass filtered from 2 Hz to 100 Hz and notch filtered between 48 Hz and 52 Hz to remove artifacts and power line interference. All electrode impedances were kept below 30 kΩ during the experiments. Electromyographic (EMG) electrodes were placed on the rectus femoris and biceps femoris of each leg to avoid the influence of EMG. EEG and EMG were both acquired by a g.HIamp system at a sampling rate of 1200 Hz. The differential voltage between electrode pairs on each leg was calculated to obtain a bipolar measure of muscle activity. During the experiments, the participants were closely observed through a video camera.

### The Experimental Paradigm

**Fig 1:**
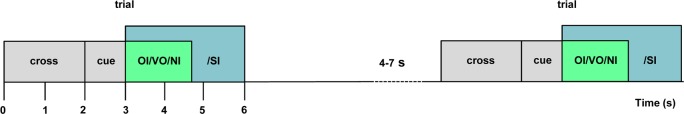
The experimental paradigm. At *t* = 0 s, a fixation cross is presented followed by a cue at *t* = 2 s. The cue stays until *t* = 3 s. Participants performs OI, NI, and VO from *t* = 3 s to *t* = 4.7 s. SI lasts from *t* = 3 s to *t* = 6 s.

The experiments were conducted in a dark and electrically shielded room. Participants were comfortably seated by a desk, the tip of their nose approximately 1 m from the computer screen. Participants were instructed to look at the center of the computer screen in front of them, to follow the cue, and to minimize eye movement artifacts. At *t* = 0 s, a cross was presented on the computer screen and remained for two seconds. At *t* = 2 s, an arrow that pointed to the left or to the right in accordance with the direction of leg movement imagery, appeared on the screen at random and lasted for 1 s. Participants were asked to perform motor observation or imagery of their legs from *t* = 3 s and stop at the appearance of “end” on the screen. The next trial appeared at a random interval of 4 s to 7 s subsequently. A presentation of the videos and their reversals were controlled by the psychophysics toolbox 3.0 [19]. Fig 1 shows the trial of the experiments.

Observation and motor imagery were separated into different runs. Participants performed MI under three conditions, called “object-oriented imagery” (OI), “non-object-oriented imagery” (NI), and “simple imagery” (SI). In these cases, participants were instructed to perform leg flexion-extension kinesthetic imagery. Besides, participants were required to watch the leg movement under “visual observation” (VO) condition.

**Fig 2:**
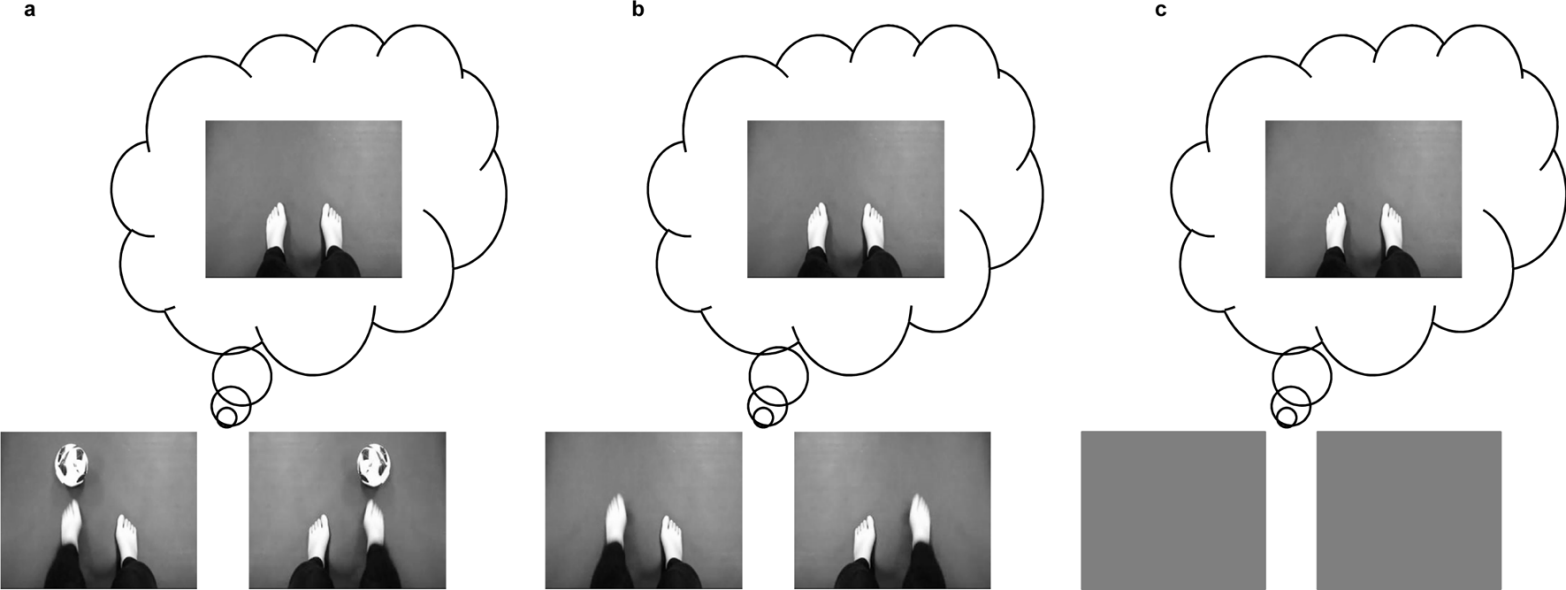
Three different tasks: (a) imagining a leg flexion-extension under the object stimulus (OI); (b) imagining a leg flexion-extension without goal (NI); (c) imagining a leg flexion-extension without the stimulus (SI).

Under the OI and VO conditions, a video concerning a unilateral leg kicking the ball (Fig 2(A)) was presented at the center of the screen from *t* = 3 s to *t* = 4.7 s. The direction of the video was the same as the cue presented at *t* = 2 s. Under the NI condition, there was also a video (Fig 2(B)) displaying the same flexion-extension movement but without the object. The length of the videos was the same for OI and NI. Under the OI and NI conditions, participants were asked to watch the leg and imagine a flexion-extension movement from *t* = 3 s to *t* = 4.7 s, while during this period under the VO condition, participants were instructed to watch this movement. Under the SI condition, both of the videos were removed and participants were required to imagine a `flexion-extension movement once. During this period, a blank screen was presented from *t* = 3 s to *t* = 6 s (Fig 2(C)).

Each run of OI, VO and NI comprised five trials for the left and five trials for the right leg. Each run of the SI comprised four trials for the left and four trials for the right leg. There were in total 12 runs for OI, VO and NI, and 15 runs for SI. The order of the different types of runs was randomized.

### Preprocessing and Feature Extraction

The EMG was checked to remove EEG trials with a fluctuation in EMG. After visually checking, approximately 9.1% trials were excluded from all further analyses. An EMD-regression analysis [20] was employed to reduce the influence of eye movements on the EEG. To reduce the impact of the choice of zero reference, researchers have studied and tried to find new approaches [21,22]. In this study, for reducing the impact of volume conduction and the choice of zero reference, improving spatial resolution and increasing the signal-to-noise ratio of EEG, the surface Laplacian [23] was used to obtain the information beneath the electrodes. For example, the EEG data at Cz can be estimated as:

Cz=Cz-0.25(FCC1h+FCC2h+CCP1h+CCP2h)

**Fig 3:**
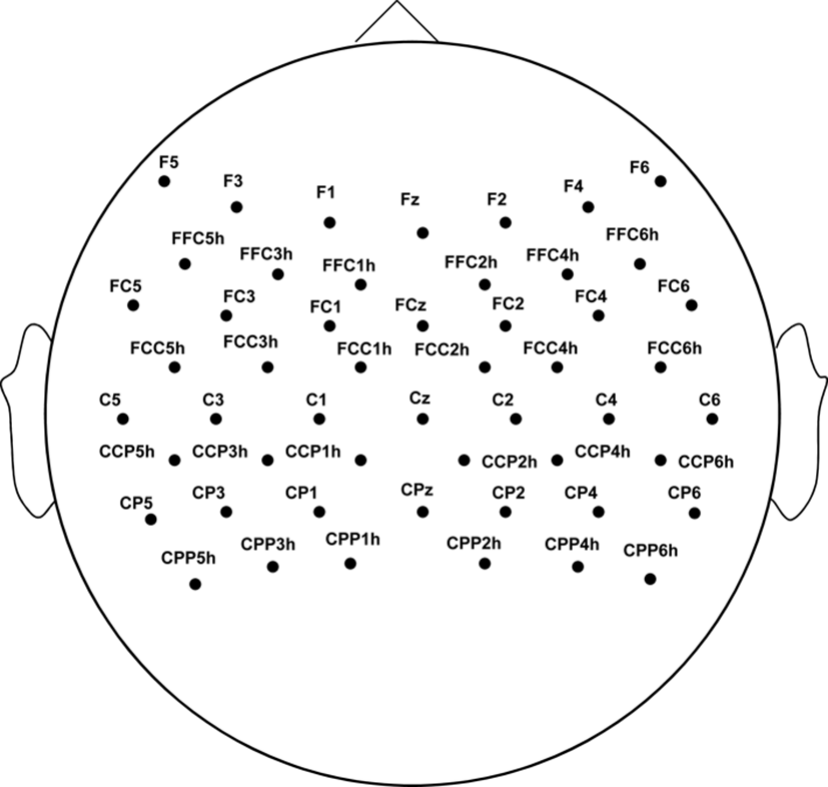
Distribution of the electrodes that are analyzed on the motor area.

The ERD/ERS spectra topographical view [24] was calculated based on fast Fourier transform, and the reference period was 1 s, from *t* = 0.5 s to *t* = 1.5 s in each trial. This study focused on the changes of the motor area, and the electrodes (Fig 3) located on this area were selected for further analysis. The suppression index was calculated as the ratio of the average power during each condition relative to the power during the reference period for quantitative analysis. The suppression index, which is less than zero, indicates suppression in the EEG amplitude (ERD), whereas the suppression index, which is greater than zero, indicates enhancement in the EEG amplitude (ERS). The suppression index, which was the quantification of the level of ERD/ERS, was used as an indicator of the ability of MI.

### Statistical Analysis and Verification

Since the EEG before *t* = 3 s is irrelevant to the four conditions, only the period of kinesthetic imagery or observation was analyzed. It is found that unilateral motor imagery provides suppression on motor related rhythms over the primary sensorimotor area, and this suppression only appears over the contralateral hemisphere [25]. Therefore, the suppression index of the imagery or observation process over the contralateral hemisphere was used as an indicator to analyze by one-way analysis of variance (ANOVA). It was performed to confirm the difference between the four conditions. To evaluate the ability of MI in every condition, the suppression indexes at the contralateral and ipsilateral hemispheres were analyzed by one-way ANOVA.

To explore the relationship of EEG between electrodes over the primary sensorimotor area, a Granger causality analysis was applied. It is a statistical measure based on the concept of time series forecasting. Specifically, if the current state of a time series is better predicted by incorporating the past knowledge of a second series, the second series is said to have a causal influence on the first [26]. The current study used the implementation via multiple vector autoregressive (MVAR) modeling with the assistance of the Granger causal connectivity analysis Matlab toolbox, which was detailed in [27]. The model order is an important parameter, which is the number of time-lags (*p*). Too few lags can lead to a poor representation of the data, whereas too many can lead to problems of model estimation. The method to specify the model order is to minimize a criterion that balances the variance accounted for by the model, against the number of coefficients to be estimated. Two criteria are implemented: the Akaike information criterion (AIC) and the Bayesian information criterion (BIC). For *n* variables:

$$\text{AIC}\left(p\right)=\text{ln}\left(\text{det}\left(\Sigma \right)\right)+2pn^{2}/T$$ (1)

$$\text{BIC}\left(p\right)=\text{ln}\left(\text{det}\left(\Sigma \right)\right)+\text{ln}\left(T\right)pn^{2}/T$$ (2)

where Σ represents the noise covariance matrix of the unrestricted model, and *T* is the length of data. In cases where the model order specified by the minimal BIC/AIC is too large to permit feasible computation, or in cases where the BIC/AIC does not reach a clear minimum over the range tested, a smaller model order can be chosen on condition that the BIC/AIC shows no further substantial decreases at higher orders [27]. All calculations were carried out in Matlab.

## Results

**Fig 4:**
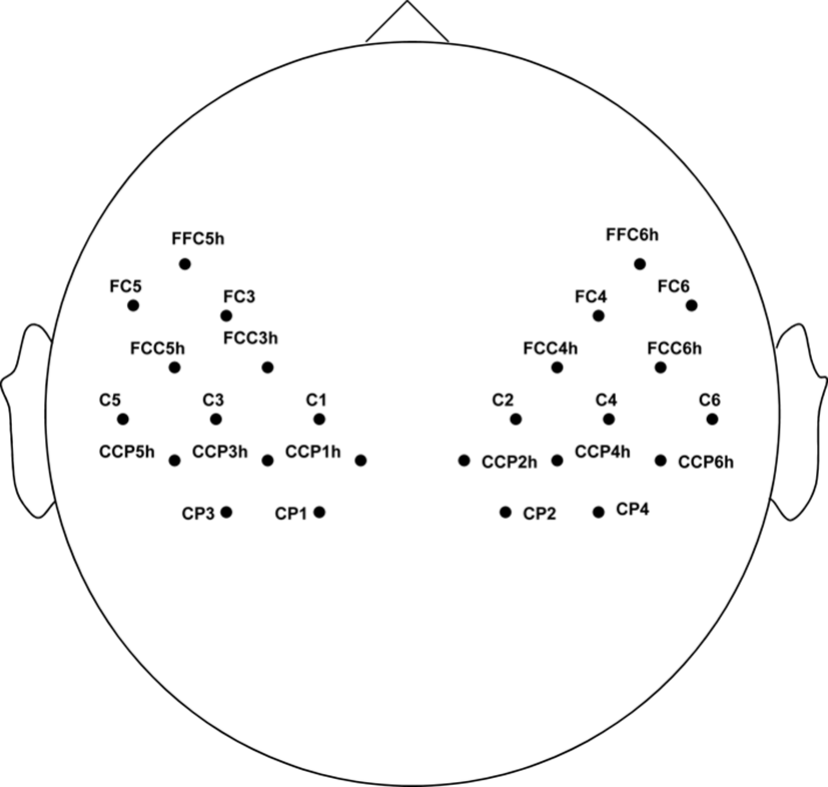
Distribution of the lateral electrodes where EEG is suppressed under OI and NI conditions.

The EEG data of four conditions in mu (8–12 Hz) and beta (18–22 Hz) rhythms, which were the common frequency ranges related to motor imagery [14,28], was analyzed. Mu and beta rhythms were suppressed during all four conditions. Whereas under the OI and NI conditions, the locations of the electrodes where EEG was suppressed and two hemispheres were significant difference (by t-test (*p* < 0.05)), were on the lateral area. The electrode distribution is illustrated in Fig 4.

To confirm the difference between the four conditions, a one-way ANOVA analysis was used by the suppression index of mu and beta rhythms on contralateral electrodes as shown in Fig 4. In mu rhythm, the significant differences were found between OI and NI (*F* (1, 26) = 19.3, *P* < 0.05), and between OI and SI (*F* (1, 26) = 6.28, *P* < 0.05). Furthermore, there was a significant difference between OI and VO (*F* (1, 26) = 22.13, *P* < 0.05) in beta frequencies. In addition, a weak significant difference was found between OI and NI (*P* = 0.0612) in beta frequencies.

**Fig 5:**
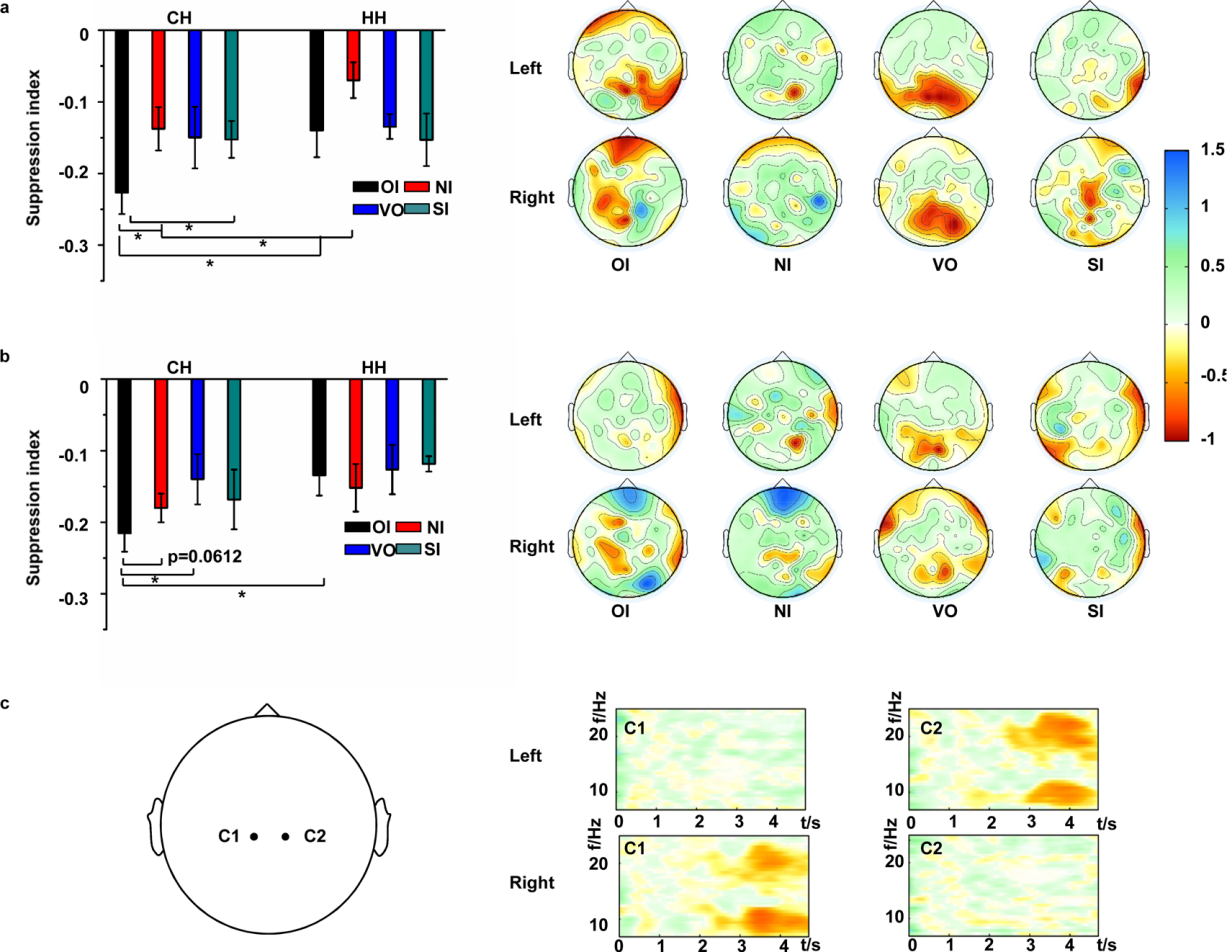
Suppression for perception conditions. (a) Statistical suppression index and topographical views in mu rhythm. (b) Statistical suppression index and topographical views in beta rhythm. (c) ERD/ERS maps of OI on subject 3 at C1 and C2. The X axis indicates time (seconds). Participants image from *t* = 3 s to *t* = 4.7 s. The Y axis indicates frequency (Hz). CH and HH indicate contralateral and ipsilateral hemispheres. Left and right indicate the direction of the cue at *t* = 2 s.

To evaluate the ability of MI under every condition, suppression indexes at contralateral and ipsilateral hemispheres were studied by using one-way ANOVA. The results demonstrated that the unilateral leg motor imagery produced a significant difference between two hemispheres (mu: *F* (1, 26) = 16.93, *P* < 0.05; beta: *F* (1, 26) = 23.54, *P* < 0.05) under the OI condition. For the NI condition, significant difference was also found in mu rhythm (*F* (1, 26) = 17.69, *P* < 0.05). Fig 5(A) shows the statistical suppression indexes for the four conditions over contralateral and ipsilateral hemispheres, and the topographical distributions of the four conditions in mu rhythm. Each head depicts the difference between baseline and imagery or observation. The upper panel of topographical distributions illustrates the left cue, and the other panel indicates the right cue. The distributions showed that under the OI condition, suppression in the mu rhythm was evident on the lateral area, while it was evident on the central area under the SI and VO conditions. Fig 5(B) shows the statistical suppression index for the four conditions at contralateral and ipsilateral hemispheres, and the topographical distributions of the four conditions in beta rhythm. The distributions of topographical views are the same as Fig 5(A). In the Fig 5(B), there was evident beta suppression for the OI condition. The ERD/ERS maps of subject 3 for OI are shown in Fig 5(C) on C1 and C2.

**Fig 6:**
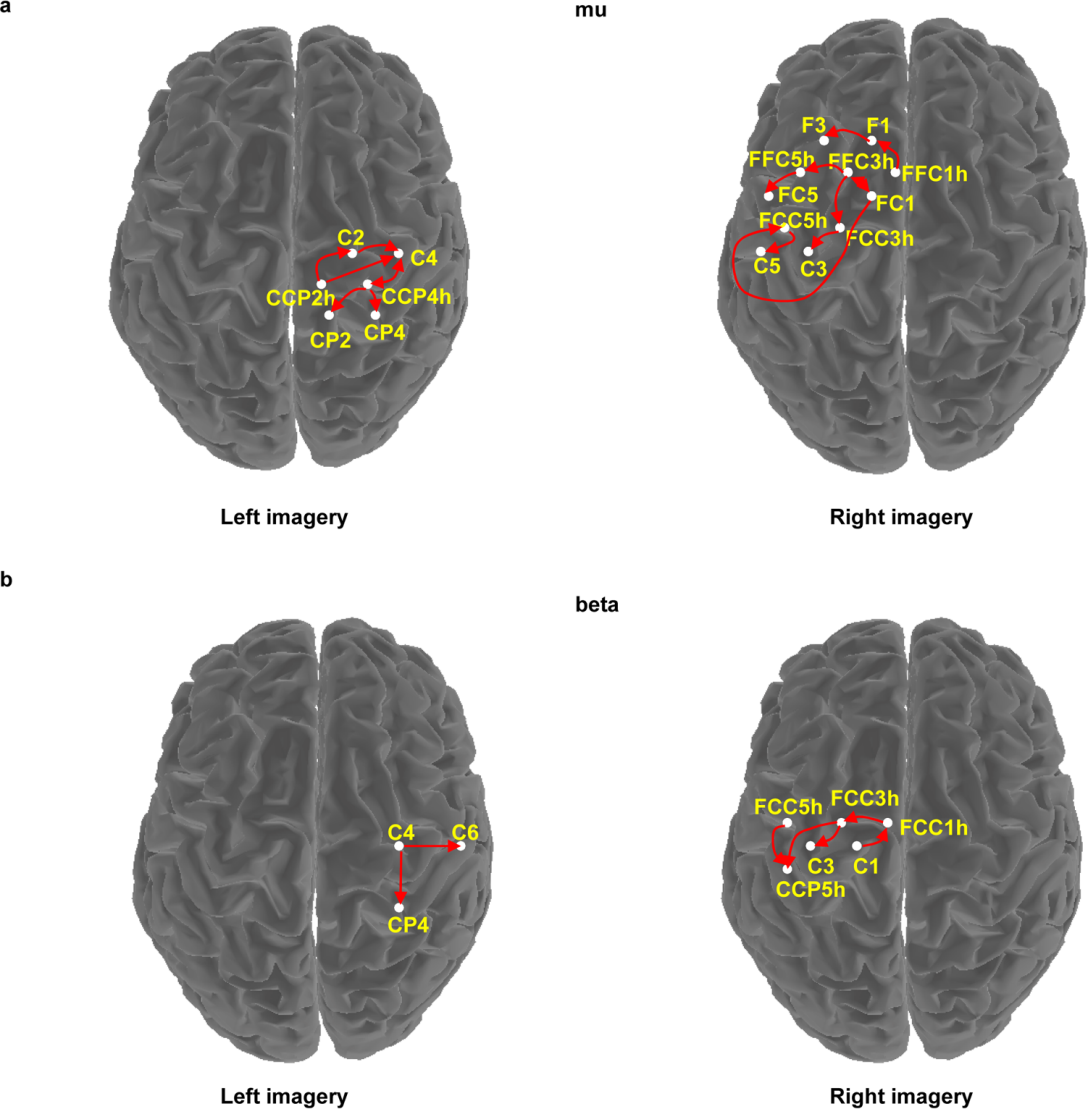
EEG connectivity model. (a) The connectivity in the mu rhythm. (b) The connectivity in the beta rhythm. The direction of the arrow indicates EEG causal flow direction. The connectivity is significant (*P* < 0.01).

Studies show the leg functional area concentrated on the central area [29]. In the present study, obvious mu and beta suppressions were found on the lateral area under OI, as mentioned in [30,31]. To study the relationship between the leg functional area and lateral area, a Granger causal connectivity analysis over the motor area was needed. The connectivity of mu and beta frequencies was studied during the object-oriented kinesthetic imagery. As the EEG before *t* = 3 s is irrelevant to motor imagery, only the period of kinesthetic imagery was analyzed. The results displayed in vertical view are shown in Fig 6. The connectivity between pairs of electrodes connected with an arrow line was significant (*P* < 0.01). The EEG was coupled in the way that the output of the supplementary motor area electrodes fed into the primary motor area electrodes and the output of the electrodes near the central area (Cz) where mu and beta frequencies originated [32] fed into the lateral area electrodes.

## Discussion

The results of this study demonstrated that the object-oriented motor imagery was significantly different from other conditions. Stronger mu suppression in object-involved motor imagery than in other kinesthetic imagery conditions could be found over the contralateral hemisphere. That is, when an object or a goal is involved in the imagery, the amplitude of EEG oscillations in mu rhythm will be obviously reduced, suggesting greater somatosensory and motor system involvement. Moreover, mu rhythm or alpha frequencies (8–13 Hz) can index the mirror neuron system or attentional mechanisms [14]. An object may make the participants more involved in the imagery. Therefore, this result demonstrates that mirror neurons or attentional mechanisms are more affected by the participants’ active involvement in the imagery. Furthermore, there was a significant mu difference between contralateral and ipsilateral hemispheres under the NI and OI conditions, while the difference was not significant for SI. Namely, vivid stimulus may improve the ability of MI. Besides, the human motor neurons is also mediated by the mirror neuron system during observation [33]. The present data of the VO condition suggested that mu rhythm was suppressed under the observation condition, and there was no significant mu suppression difference between OI and VO at the contralateral hemisphere. This result indicates that mirror neuron system participates in both of the conditions. Under the OI condition, there was a significant mu difference between contralateral and ipsilateral hemispheres, but the difference under the VO condition was not significant. Thus, when imagery is executed during the observation, mirror neuron systems will be greater involved.

Beta oscillation is a kind of motor related rhythm. Babiloni et al. [12] indicate beta suppression on the contralateral area during the finger movement. Similarly, Neuper and Pfurtscheller [28] also discover this in a hand movement experiment. Park suggests that the beta suppression is calibrated to cognitive factors [34], whereas Neuper and Pfurtscheller [28] consider that the beta frequency band is strongly related to motor behavior on the motor area. In the present study, beta suppression under the OI condition was found over contralateral hemisphere. It was significantly different from the suppression of VO and a weak significant difference with the suppression of NI. Moreover, the beta suppressions of contralateral and ipsilateral hemispheres were significantly different under the OI condition, but this difference was not significant under other conditions. These results demonstrate that an object involved in the imagery may improve the ability of MI in the beta frequencies.

The connectivity of motor area indicated that the supplementary motor area had a causal relationship with the primary motor area. This demonstrates that primary motor area is involved in the motor control as mentioned in the studies about connectivity of motor areas [35–37]. The study results of connectivity presented significant connectivity between the lateral and central areas. That is, EEG on the lateral area had a causal relationship with the central area. This result reveals that the suppressions of mu and beta rhythms on the lateral area are affected by the suppressions on the central area. According to the results of connectivity on cues, the right-handed participants presented a more extensive connectivity in the dominant hemisphere. This result is consistent with the study [38] about the connectivity of human motor cortex which indicates that the connectivity is asymmetric and more extensive in the dominant hemisphere.

To conclude, this study has explored the effect of an object on kinesthetic motor imagery by motor related rhythms, indexing a possible mechanism of the mirror neuron system, perceptual attentional mechanism and the ability of motor imagery. We found that the mirror neuron system and perceptual attentional mechanism are affected by an object in the imagery. Moreover, an object-oriented motor imagery can improve the ability of motor imagery. Results of this study might be favorable for motor rehabilitation of lower extremities and motor skills training based on motor imagery.
